# The deubiquitinating protein OTUD6B promotes lung adenocarcinoma progression by stabilizing RIPK1

**DOI:** 10.1186/s13062-024-00489-8

**Published:** 2024-06-16

**Authors:** Miaomiao Yang, Yujie Wei, Xin He, Changwei Xia

**Affiliations:** 1 Department of Nephrology, Henan Provincial Key Laboratory of Kidney Disease and Immunology, Henan Provincial Clinical Research Center for Kidney Disease, Henan Provincial People’s Hospital and People’s Hospital of Zhengzhou University, 7 Weiwu Road, Zhengzhou, 450053 Henan China; 2 Heart Center of Henan Provincial People’s Hospital, Central China Fuwai Hospital of Zhengzhou University, Fuwai Central China Cardiovascular Hospital and Central China Branch of National Center Fuwai Cardiovascular Diseases, Zhengzhou, China; 3 National Engineering Laboratory for Internet Medical Systems and Applications, The First Affiliated Hospital of Zhengzhou University, Zhengzhou University, No. 1 Jianshe Road, Erqi District, Zhengzhou, 450052 Henan China; 4 Department of Clinical Laboratory, The Fifth Affiliated Hospital of Zhengzhou University, No.3 Kangfuqian Street, Erqi District, Zhengzhou, China

**Keywords:** Deubiquitinating protein, Lung adenocarcinoma, Tumor progression, OTUD6B, RIPK1

## Abstract

**Background:**

There is growing evidence indicating that deubiquitinating enzymes may contribute to tumor progression and can serve as promising therapeutic targets.

**Methods:**

The overexpression of deubiquitinase OTUD6B in lung adenocarcinoma (LUAD) and its adjacent tissues was analyzed by immunohistochemistry and TCGA/GO database. Survival analysis further supported OTUD6B as a potential target for LUAD treatment. We assessed the effect of OTUD6B on LUAD cell growth using cell viability assays and conducted TUNEL staining, migration, and invasion experiments to investigate the impact of OTUD6B on the apoptosis and metastasis of LUAD cells. Additionally, we established a transplanted tumor model in nude mice to validate our findings in vivo. Finally, using IP mass spectrometry and co-IP experiments, we screened and confirmed the influence of RIPK1 as a substrate of OTUD6B in LUAD.

**Results:**

OTUD6B is highly overexpressed in human LUAD and predicts poor prognosis in LUAD patients. OTUD6B knockdown inhibited the proliferation of LUAD cells and enhanced apoptosis and inhibited metastasis in LUAD cells suppressed. A549 xenografts revealed that OTUD6B deletion can slow down tumour growth. Additionally, OTUD6B can bind to RIPK1, reduce its ubiquitination level and increase its protein stability.

**Conclusions:**

Our results suggest that OTUD6B is a promising clinical target for LUAD treatment and that targeting OTUD6B may constitute an effective anti-LUAD strategy.

**Supplementary Information:**

The online version contains supplementary material available at 10.1186/s13062-024-00489-8.

## Background

Lung cancer is the most prevalent cancer and one of the leading causes of cancer death worldwide [1]. Non-small cell lung cancer (NSCLC), specifically lung adenocarcinoma (LUAD), accounts for nearly 80% of lung cancer cases. Despite advances in technology and treatment, the five-year overall survival rate for LUAD patients remains poor, with unsatisfactory recurrence rates [2]. As such, it is critical to further understand the prognosis of LUAD patients to identify new therapeutic targets and improve survival rates.

Ubiquitination and deubiquitination are crucial processes for regulating protein stability, metabolism, and control of physiological and pathological processes in cells [3, 4]. Deubiquitinases (DUBs) from the ovarian tumor (OTU) family play a significant role in these processes. The primary function of these enzymes is to remove ubiquitin molecules from substrates, thereby preventing their degradation by the proteasome. Additionally, OTU family members have been found to regulate the stability and activity of several proteins involved in cellular signaling [5, 6].

One extensively studied member of the OTU family is OTUB1, which has been shown to regulate the activity of various proteins, including the tumor suppressor protein p53 and the transcription factor HIF-1α. Through deubiquitination, OTUB1 promotes the stability of p53, thereby enhancing its tumor suppressor activity. Similarly, deubiquitination of HIF-1α promotes its stability and enhances its transcriptional activity [7, 8].

OTUD5, a member of the OTU family, has been shown to contribute to tumor progression [5]. In tumor cells, OTUD5 promotes tumor progression by deubiquitinating TRIM25 [9]. In addition, OTUD5 can also mediate YAP deubiquitination in macrophages and subsequently promote M2 phenotype polarization, which facilitates the progression of triple-negative breast cancer [10]. Additionally, researchers have reported that OTUD5 may play an essential role in both antiviral and antitumor immunity [11].

Overall, dysregulation of OTU family members has been implicated in various diseases, including cancer, inflammatory disorders, and neurodegenerative diseases, emphasizing their potential as therapeutic targets [5, 12, 13].

OTUD6B is another member of the OTU family of DUBs that has been linked to the regulation of several cellular processes contributing to cancer progression, including cell proliferation, apoptosis, and metastasis [14–16]. While the antitumor effects of OTUD6B have been reported in multiple cancers, such as breast, liver, and pancreatic adenocarcinomas, its expression in LUAD and impact on patient prognosis have yet to be established. Moreover, the potential substrates of OTUD6B in LUAD have yet to be elucidated. Hence, this study aimed to demonstrate the inhibitory effect of low OTUD6B expression on LUAD through in vivo and in vitro experiments and to screen potential active substrates of OTUD6B and RIPK1 using mass spectrometry. Finally, we analyzed the correlation between OTUD6B and RIPK1 in LUAD. Our findings suggest that targeting OTUD6B may be a potential anti-LUAD strategy, as it has been shown to inhibit LUAD growth.

## Methods

### Cell culture

The human LUAD cell lines H1395 and A549 were obtained from the Chinese National Infrastructure of Cell Line Resources and cultured in DMEM (VivaCell) supplemented with 10% fetal bovine serum (FBS; Gibco) and 100 U/ml penicillin/streptomycin (Corning) at 37 °C in a humidified atmosphere with 5% CO2.

### Patients and specimens

The LUAD tissue microarray (TMA) cohort included 119 samples of LUAD tissues and corresponding normal tissues, all of which were procured from the First Affiliated Hospital of Zhengzhou University located in Zhengzhou, China. To ensure ethical compliance, our study obtained approval from the Ethics Committee of the hospital, and all participating patients provided written informed consent before their surgery.

### Immunohistochemical (IHC) staining

In brief, tissue array sections (4 μm) or paraffin-embedded tissue sections were dehydrated, and peroxidase activity was blocked. Antigen recovery was achieved using 0.01 mol/L citric acid buffer (pH 6.0) and a pressure cooker. The tissues were then incubated with primary antibodies overnight at 4 °C, followed by staining with tissue staining kits (SP-9000) and 3,3′-diaminobenzidine tetrahydrochloride (DAB) (ZLI-9032; ZSGB-BIO, Beijing, China). Hematoxylin and eosin (HE) were used to counterstain the slides. The stained glass slides were observed, and images were obtained through a microscope. The sections were scored according to cell staining intensity and range of positive cells, and then multiplied to obtain the final score.

1. Cell staining intensity score: Slices are observed under a microscope and scored according to the depth of cell staining. For example, cells that are not colored are negative and score 0; Light yellow cell is weak positive, 1 point; Cells with medium yellow or brown color without background coloring, or cells with dark brown color but light brown background were moderately positive, 2 points were scored; Cells with dark brown or tan color and no background coloring are strongly positive, score 3 points. 2. Positive cell expression number score: The sections were observed under a low-power microscope to evaluate the percentage of positive cells in the total number of cells. Score according to the percentage of positive cells, for example, positive cells = 0%, 0 points; 0% < number of positive expression cells < 25%, score 1; 25% < the number of positive expression cells < 50%, 2 points; 50% < the number of positive expression cells < 75%, 3 points; The number of positive expression cells > 75% was recorded as 4 points.

### Acquisition and processing of LUAD datasets

Gene expression and survival data from 4 LUAD datasets (GSE19188, GSE31210, GSE37745, and GSE43767) were obtained from the Gene Expression Omnibus (GEO) database (http://www.ncbi.nlm.nih.gov/geo/). The TCGA-LUAD dataset and corresponding clinical data were downloaded from The Cancer Genome Atlas (TCGA) repository (https://tcga-data.nci.nih.gov/tcga/).

### Quantitative real-time PCR (qPCR)

Total RNA was extracted from LUAD cells using TRIzol reagent (Invitrogen Life Technologies) following the manufacturer’s protocol. The RNA concentration and purity were assessed with a NanoDrop 2000 (Thermo Scientific). First-strand cDNA was synthesized from 1 μg of total RNA using the PrimeScript RT Reagent Kit with gDNA Eraser (TaKaRa). Briefly, 1 μL of gDNA Eraser, 2 μL of 5gDNA Eraser buffer, and RNase-free dH2O were added to 1 μg of total RNA and incubated at 42 °C for 2 min. The enzyme mixture was subsequently added, and the mixture was incubated at 37 °C for 15 min. Quantitative real-time PCR was performed using SYBR Premix Ex Taq II (Roche) in an Agilent Mx3005P. The PCR amplification protocol consisted of 40 cycles at 95 °C for 30 s, 95 °C for 5 s, and 60 °C for 30 s. The abundance of mRNA for each gene of interest was normalized to that of GAPDH. The data were analyzed using the 2^−ΔΔCt^ method. The sequences of primers used were as follows: OTUD6B, forward 5′-TGAGAAGGCATCGCAAAGAGA-3′ and reverse 5′-ATCTTCGGTGAGTTGCTTCCT-3′; RIPK1, forward 5′-GGGAAGGTGTCTCTGTGTTTC-3′ and reverse 5′-CCTCGTTGTGCTCAATGCAG-3′; and GAPDH, forward 5′-AAAGGGTCATCATCTCTG-3′ and reverse 5′-GCTGTTGTCATACTTCTC-3′.

### Western blotting

After LUAD and paracancerous tissues were minced or cells were collected, they were lysed with RIPA lysis buffer (Beyotime) for Western blot analysis. All primary antibodies were diluted and incubated overnight at 4 °C. The secondary antibodies, peroxidase-conjugated goat anti-mouse IgG and peroxidase-conjugated goat anti-rabbit IgG, were purchased from ZGSB-Bio, Inc. (Beijing, China). The membranes (PVDF) were incubated with the corresponding secondary antibodies for 2 h at 25 °C, after which the proteins were detected using an enhanced chemiluminescence (ECL) kit (Beyotime).

### Antibodies

Primary antibodies used in this study include anti-Myc (IP: 1:50, #AG0305, Beyotime Biotechnology), anti-Ubiquitin (Ub) (IP: 1:100, #sc-166553, Santa Cruz Biotechnology), anti-Flag (IP:1:100, #AG8050, Beyotime Biotechnology), anti-HA (IP:1:100, #AG8057, Beyotime Biotechnology, Jiangsu, China), anti-OTUD6B (WB: 1:1000, IP: 1:100, IHC:1:100, #PA5-118135, #PA5-146415, ThermoFisher Scientific), anti-RIPK1 (WB: 1:1000, IP: 1:100, #A7414, ABclonal), (IHC:1:100, #PA5-20811, ThermoFisher Scientific), anti-β-actin (WB: 1:20000, IP:1:100, #66009-1-Ig, Proteintech), Anti-GAPDH (WB: 1:2000, #ab245355, abcam), Anti-Ki-67(IHC:1:100, # MA5-14520, ThermoFisher Scientific). Secondary antibodies include goat anti-mouse lgG (#2B-2301) and goat anti-rabbit lgG (#2B.6482305) (Beijing Zhongshan Jingiao Biotechnology), normal mouse IgG (#sc-2025, Santa Cruz Biotechnology) and EasyBlot anti-mouse lgG(HRP) (#GTX221667-01, Genetex).

### Cell viability and clonogenic survival assay

The untreated LUAD cell line and the stably knocked down or overexpressed LUAD cell line were seeded uniformly in a 96-well plate at a density of 3 × 10^3^ cells per well and cultured continuously for 5 days. Cell viability was assessed daily using a Cell Counting Kit-8 (CCK-8) (Beyotime Biotechnology, China) according to the manufacturer’s instructions. For colony formation assays, LUAD cell lines from different treatment groups were seeded into 6-well plates at a density of 500 cells per well in triplicate and incubated for 10 days. The colonies were fixed with 4% paraformaldehyde (Solarbio, Beijing, China) and stained with crystal violet (Solarbio, Beijing, China) for subsequent statistical analysis.

### EdU cell proliferation assay

To perform EdU proliferation assays, adherent cells were washed three times with PBS and digested with trypsin. Next, 5 × 10^4^ cells were seeded into each well of a 24-well plate and incubated at 37 °C for 12–16 h. An appropriate amount of 35–50 μM EdU medium was prepared using complete medium. The medium in the 24-well plate was replaced with prepared EdU medium, and the plate was incubated at 37 °C for 2 h. The medium was then discarded, and the cells were washed twice with 0.5 ml of PBS for 3–5 min each. Next, 0.3–0.4 ml of 4% paraformaldehyde solution was added to each well of the 24-well plate, and the cells were fixed for 30 min at room temperature. The fixative was then discarded, and 0.25–0.35 ml of 2 mg/mL glycine solution was added to each well. The plate was shaken for 5 min at room temperature, after which the decolorization solution was discarded. The cells were washed 3 times with 0.5 ml of PBS per well. The prepared penetrating agent was added to each well of the 24-well plate according to the kit instructions, and the plate was shaken for 5 min for decolorization and washing. Next, 0.2 mL of 1 × Apollo® Staining Solution was added to each well, the plate was covered with aluminum foil, and the plate was incubated for 30 min on an orbital shaker at room temperature. After that, 0.3 mL of a preprepared osmotic agent was added to each well, and the plate was incubated for 10 min on a shaker. Reagent F was diluted 100 times with ddH2O to make a 1 × Hoechst 33,342 solution, and 0.2 ml of the solution was added to each well of the 24-well plate. The cells were then incubated for 30 min, washed twice with 0.5 ml of PBS for 5 min each, and imaged using fluorescence microscopy. Full nuclei were stained blue under UV light, while proliferating nuclei were stained red under green light. The EdU kit used in this study was purchased from RiboBio (Guangzhou, China).

### TUNEL cell apoptosis test

After the cells were digested with trypsin, the cells were resuspended in medium, seeded in 24-well plates at 5 × 10^4^ cells per well and incubated at 37 °C for 12–16 h. Then, the cells were fixed with 4% paraformaldehyde for 30–60 min, washed once with PBS, washed twice with PBS containing 0.1% Triton X-100, incubated in an ice bath for 2 min, and washed twice with PBS; 50 μL of TUNEL was added (the TUNEL kit was purchased from RiboBio, Guangzhou, China). The cells were incubated at 37 °C in the dark for 60 min and washed 3 times with PBS. Fluorescence microscopy was used for image acquisition and analysis (full staining of cell nuclei under UV light was blue, and apoptotic cell nuclei under green light were stained green).

### Cell invasion and migration assays

First, the Matrigel, which was previously stored at -20 °C, was removed, the mixture was stored temporarily at 4 °C, and the mixture was thawed for later use. The next day, the Matrigel was diluted with complete medium. The solution was evenly distributed in the upper chamber of the transwell chamber and temporarily stored in the incubator. Cells from each group were digested, centrifuged, resuspended in serum-free medium, and counted on a cell counting plate. Serum-free medium (0.2 ml) was added to each well of the upper chamber of the transwell chamber, and 5 × 10^4^ cells were inoculated. DMEM containing 15% fetal bovine serum was added to the lower chamber. The cells were incubated for approximately 30 h in the incubator. Then, the transwell chamber was gently clipped with forceps, and the noninoculated cells were removed from the upper layer with sterile cotton. Then, the cells were fixed with 4% paraformaldehyde for 5 min, stained with crystal violet, and washed off excess color with PBS. Finally, the cells were dried at room temperature, photographed under a microscope, and counted using ImageJ. The transwell migration experiment does not require Matrigel treatment, and cells can be directly inoculated into the upper chamber of the transwell chamber for culture.

### Mouse model of human LUAD

We selected 6-week-old female BALB/c nude mice for tumor xenograft experiments. BALB/c nude mice were injected subcutaneously with 2 × 10^6^ A549 cells, the tumor-bearing mice were randomized into groups, each group contained six mice (n = 6), tumor growth was observed, mouse weight changes were recorded, and tumor size was determined via caliper measurements. The ellipsoid volume formula was used to calculate tumor volume. Mice were photographed and weighed at sacrifice. All the mice were euthanized, and the tumors were surgically removed. Tumor tissue was collected and fixed in liquid nitrogen or 4% paraformaldehyde for subsequent analysis. Investigators were blinded to the treatment groups during the data collection and subsequent data analysis. All mouse experiments were conducted in an SPF animal care facility with ad libitum access to food and water. Animal experiments were performed in accordance with the animal experiment protocol approved by the Institutional Animal Care and Use Committee of Zhengzhou University.

### Immunoprecipitation (IP) assays

Cell lysates were prepared from the indicated cells using NP-40 lysis buffer (NP-40, 150 mM NaCl, 10 mM HEPES, pH 7.4, 0.1%). The lysates were then mixed with rabbit anti-OTUD6B antibody, rabbit anti-RIPK1 antibody, and protein G-conjugated agarose at 4 °C overnight. Beads containing affinity-coupled proteins were washed 6 times with NP-40 wash buffer and then eluted with 1 mM glycine (pH 3.0). The eluate was then mixed with sample buffer, denatured, and subjected to Western blot analysis; after washing, the bound proteins were separated via SDS‒PAGE and stained with Coomassie blue. Specific bands were analyzed by mass spectrometry.

### Statistical analysis

All the statistical analyses were performed using GraphPad Prism 8 or SPSS 21.0 (IBM Corporation, Armonk, NY, USA). For comparisons between two groups, p values were calculated using a two-sided Student’s t test. For comparisons between more than two groups, p values were calculated using analysis of variance. The relationship between the expression of OTUD6B or RIPK1 and clinicopathological features was tested by a two-sided χ2 test. Survival curves were drawn using the Kaplan–Meier method and compared by the log-rank test. *P* < 0.05 indicated that the difference was statistically significant.

## Results

### OTUD6B is highly overexpressed in human LUAD and predicts poor prognosis in LUAD patients

**Fig. 1:**
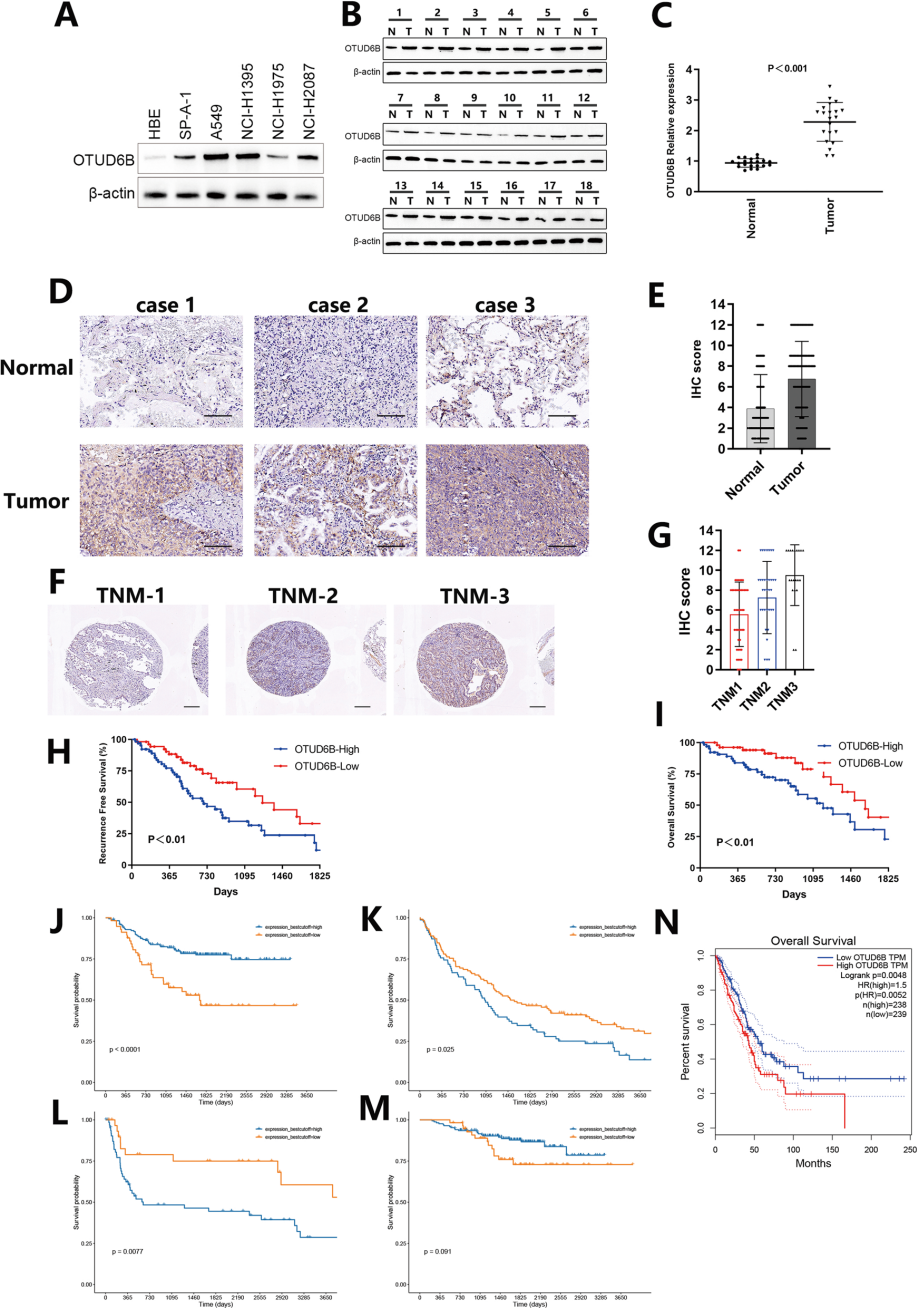
Overexpression of OTUD6B in LUAD patients predicts poor clinical prognosis. **A** The expression of OTUD6B in human bronchial epithelial-like cell lines (HBE) and human LUAD cell lines (SP-A-1, A549, NCL-H1395, NCL-H1975, and NCL-2087) was detected by Western blotting. **B** Expression of the OTUD6B protein in LUAD tissues was detected via Western blotting. N: represents adjacent nontumor tissue, T: represents LUAD tissue. **C** The expression of OTUD6B in LUAD tissues was detected by qPCR. **D**, **E** The expression and IHC score of OTUD6B in adjacent nontumor tissues and LUAD tissues from patients with different degrees of differentiation were detected by immunohistochemistry. **F**, **G** The expression of OTUD6B in LUAD tissues from patients with different degrees of malignancy was grouped according to TNM stage, and the levels of OTUD6B in the different groups were analyzed. **H**, **I** In the TMA-based cohort, Kaplan–Meier survival analysis was used to evaluate the clinical significance of OTUD6B expression for overall survival (OS) and recurrence-free survival (RFS). **J**–**M** Analysis of the expression level and survival prognosis of patients with clinical LUAD stratified by OTUD6B expression according to the GEO database. **N** The expression level and survival prognosis of LUAD patients stratified by OTUD6B expression were analyzed using the TCGA database

To investigate the expression of OTUD6B in LUAD, we examined its expression in normal human bronchial epithelial cells and various types of LUAD cell lines (Fig. 1A). Our results showed that the expression of OTUD6B was significantly greater in LUAD cell lines than in normal human bronchial epithelial cells. We also analyzed the expression of OTUD6B in LUAD and adjacent normal tissues and found that the expression of OTUD6B was greater in LUAD tissues than in adjacent normal tissues (Fig. 1B, C). Additionally, we conducted immunohistochemical staining of 119 pairs of human LUAD tissues and paraneoplastic tissue arrays to detect OTUD6B expression (Fig. 1D). The staining results of each pair of tissues were immunohistochemically scored, and we found that OTUD6B was significantly more highly expressed in cancer tissues than in paracancerous tissues (Fig. 1E). Furthermore, we analyzed the association between the IHC score of OTUD6B in patient cancer tissue and TNM stage and observed that the higher the IHC score of OTUD6B in cancer tissue was, the greater the value of TNM stage in that patient (Fig. 1F, G). These findings suggest that OTUD6B may have clinical significance in the diagnosis and prognosis of LUAD.

Subsequently, we conducted Kaplan‒Meier analysis of follow-up survival data and found that increased OTUD6B levels were negatively correlated with recurrence-free survival (RFS) and overall survival (OS) in LUAD patients (Fig. 1H, I). Additionally, we analyzed the relationships between OTUD6B expression levels and clinical characteristics and survival prognosis in LUAD patients by examining the TCGA/GEO database (Fig. 1J–N). The results of our analysis were consistent with our experimental findings. These results suggest that OTUD6B may serve as a potential prognostic biomarker for LUAD patients.

### OTUD6B knockdown inhibited the proliferation of LUAD cells

**Fig. 2:**
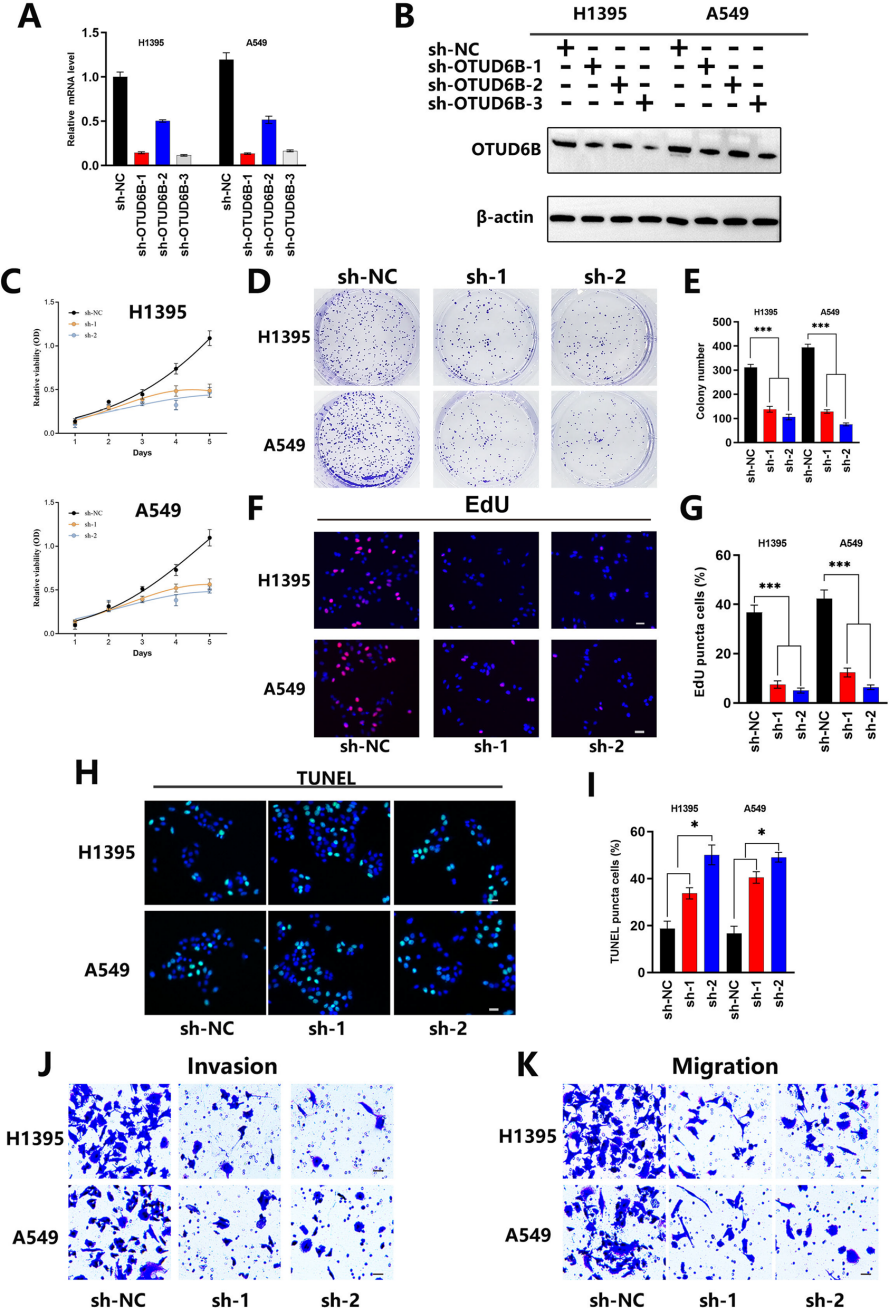
Low OTUD6B expression inhibited the proliferation and metastasis of LUAD cells. **A** The relative OTUD6B mRNA level in OTUD6B-silenced LUAD cell lines was examined via qPCR. **B** The expression of OTUD6B in LUAD cell lines with OTUD6B knockdown was detected by Western blot. **C**–**G** The proliferation of LUAD cell lines with silenced OTUD6B was examined by CCK8 proliferation, plate colony formation and EdU cell proliferation assays. **H**, **I** TUNEL staining was used to detect apoptosis in LUAD cell lines. **J**, **K** The metastasis ability of LUAD cell lines was examined by invasion and migration assays

We employed shRNA or lentiviral transduction to silence OTUD6B in the LUAD cell lines A549 and H1395 and confirmed the knockdown efficiency by qPCR and Western blot analysis (Fig. 2A, B). To investigate the effect of OTUD6B inhibition on LUAD cell proliferation, we performed CCK8, plate colony formation, and EdU cell proliferation assays. Our results revealed that knockdown of OTUD6B significantly impaired the viability, clonal expansion, and proliferation of LUAD cells (Fig. 2C–G).

### OTUD6B knockdown enhanced apoptosis and inhibited metastasis in LUAD cells

Next, we investigated the role of OTUD6B in apoptosis and metastasis. TUNEL staining revealed that knockdown of OTUD6B can induce apoptosis in LUAD cells (Fig. 2H, I). In addition, in our transwell experiments, A549 and H1395 cells transfected with shOTUD6B lacked the ability to migrate to the lower chamber, indicating that knockdown of OTUD6B expression can significantly inhibit the invasion and migration of LUAD cells (Fig. 2J, K).

### OTUD6B knockdown suppressed LUAD tumor growth in a mouse model

**Fig. 3:**
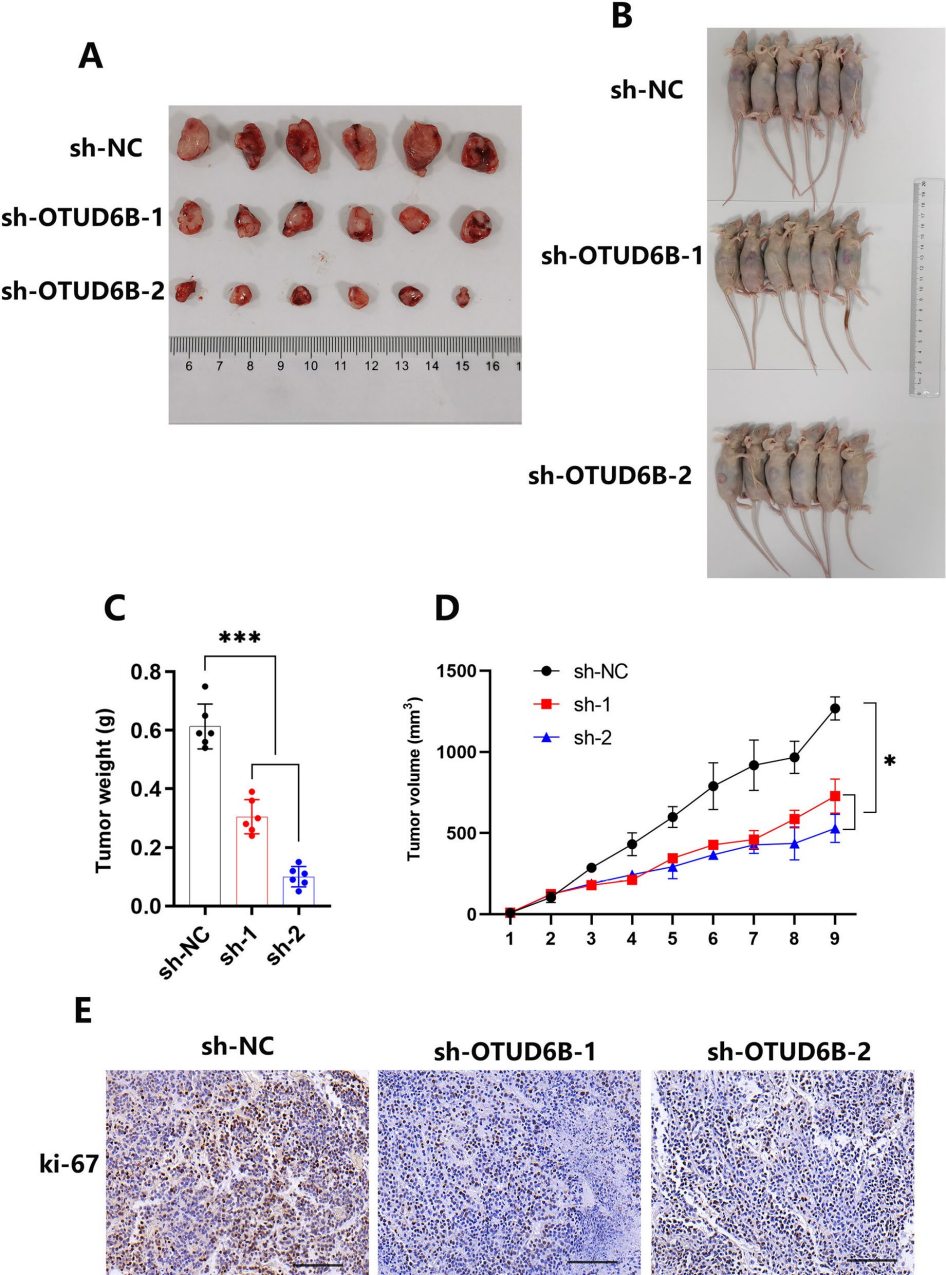
Knocking down OTUD6B inhibited LUAD tumor growth in a mouse model. **A**, **B** The sizes of tumors in the mouse model constructed from sh-NC, sh-1 and sh-2 A549 cells. **C**, **D** The volume and weight of tumors from the sh-NC, sh-1 and sh-2 A549 cell groups. **E** The expression of Ki-67 in tumor tissues was detected by immunohistochemistry. The data are presented as the means ± SD. *P < 0.05; **P < 0.01; ***P < 0.001

We subcutaneously inoculated nude mice with the normal LUAD cell line A549 (shNC) or the LUAD cell line with OTUD6B knockdown (shOTUD6B-1, shOTUD6B-2) to construct mouse LUAD models, and the tumor-bearing mice were divided into three groups (Fig. 3A, B). The results showed that the tumor growth rate of normal LUAD mice was significantly greater than that of LUAD mice with OTUD6B knockdown, as evidenced by the growth curve. We also analyzed the tumor volume and weight, which was consistent with the growth curve results (Fig. 3C, D). Then, the tumor tissues of the mice were dissected, embedded in wax blocks and sectioned, and the expression of Ki-67 in the tissues of the different groups of mice was analyzed via immunohistochemistry (Fig. 3E). The results showed that the tumor tissues of normal LUAD mice were more malignant than those of LUAD mice with knocked down OTUD6B expression.

### Overexpressing OTUD6B promotes LUAD progression

**Fig. 4:**
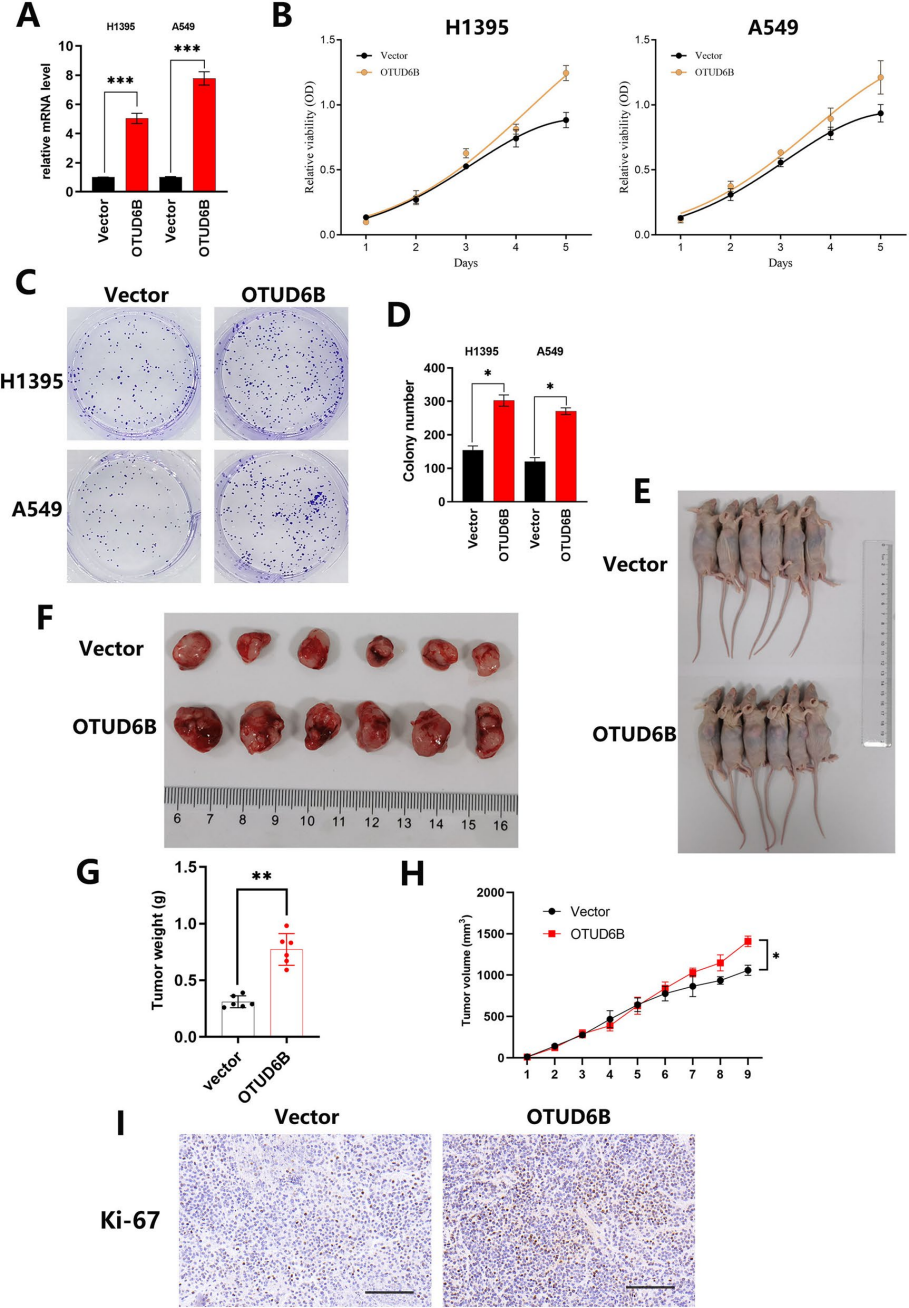
OTUD6B overexpression promotes LUAD progression. **A** The relative OTUD6B mRNA level was verified by qPCR in cells overexpressing OTUD6B and in the Kong vector-transfected LUAD cell line. **B**–**D** Cell proliferation was examined by the CCK8 assay and plate colony formation assay. **E**, **F** The sizes of tumors generated from normal LUAD cells (Vector-A549) and OTUD6B-overexpressing LUAD cells (OTUD6B-A549) in a mouse model. **G**, **H** The volume and weight of tumors generated from normal LUAD cells (Vector-A549) and OTUD6B-overexpressing LUAD cells (OTUD6B-A549) in the mouse model. **I** The expression of Ki-67 in mouse tumors was detected by immunohistochemistry. The data are presented as the means ± SD. *P < 0.05; **P < 0.01; ***P < 0.001

To further verify the effect of OTUD6B on LUAD progression, we constructed a LUAD cell line overexpressing OTUD6B and verified its expression by qPCR (Fig. 4A). Then, through CCK8 proliferation and plate colony formation experiments, we found that overexpressing OTUD6B in LUAD cell lines significantly promoted proliferation and colony formation (Fig. 4B–D). In addition, we subcutaneously inoculated normal LUAD cells (vector-A549) and OTUD6B-overexpressing LUAD cells (OTUD6B-A549) into nude mice to construct subcutaneous tumor models (Fig. 4E, F). The results showed that mice inoculated with OTUD6B-overexpressing LUAD cells (OTUD6B-A549) had significantly faster tumor growth and greater tumor weight than did those in the normal group (Fig. 4G, H). The tumor tissues of the mice were dissected, embedded in wax blocks and sectioned, and the expression of Ki-67 in different groups of mouse tissues was analyzed by immunohistochemistry. The results showed that tumors from mice inoculated with OTUD6B-overexpressing LUAD cells (OTUD6B-A549) were more malignant (Fig. 4I).

### OTUD6B can bind to RIPK1, reduce its ubiquitination level and increase its protein stability

**Fig. 5:**
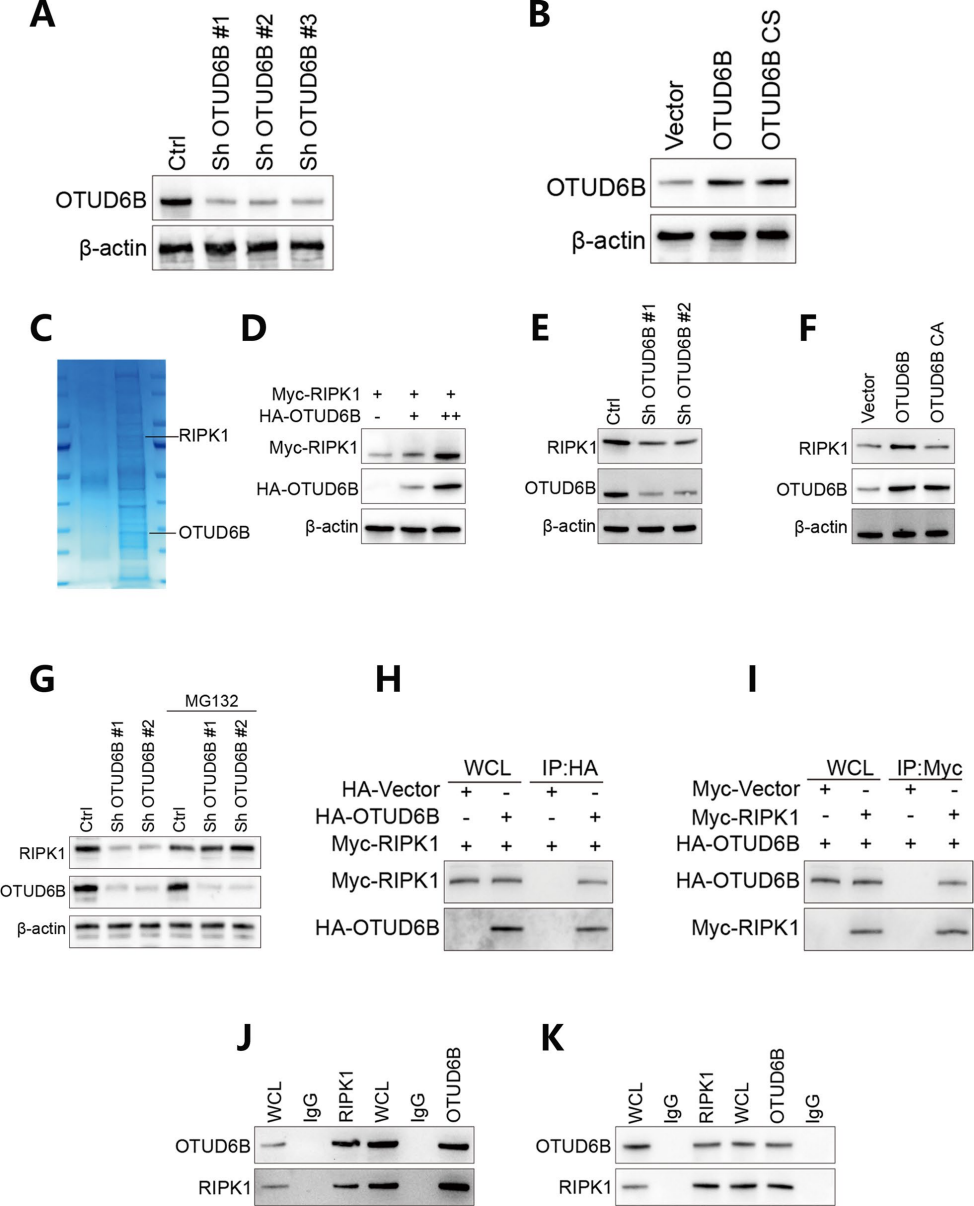
The interaction between OTUD6B and RIPK1 was verified. **A**, **B** OTUD6B was knocked down or overexpressed in LUAD cell lines, as verified by Western blotting. **C**, **D** RIPK1 interacted with OTUD6B in LUAD cells, as shown by co-immunoprecipitation experiments. **E**, **F** OTUD6B regulates the expression of RIPK1 at the protein level, as verified by Western blotting. **G** A proteasome pathway inhibitor (MG132) was used to evaluate the effect of interfering with OTUD6B on the degradation of RIPK1. **H**–**K** The interaction between OTUD6B and RIPK1 was verified by two-way coimmunoprecipitation of exogenous and endogenous proteins

**Fig. 6:**
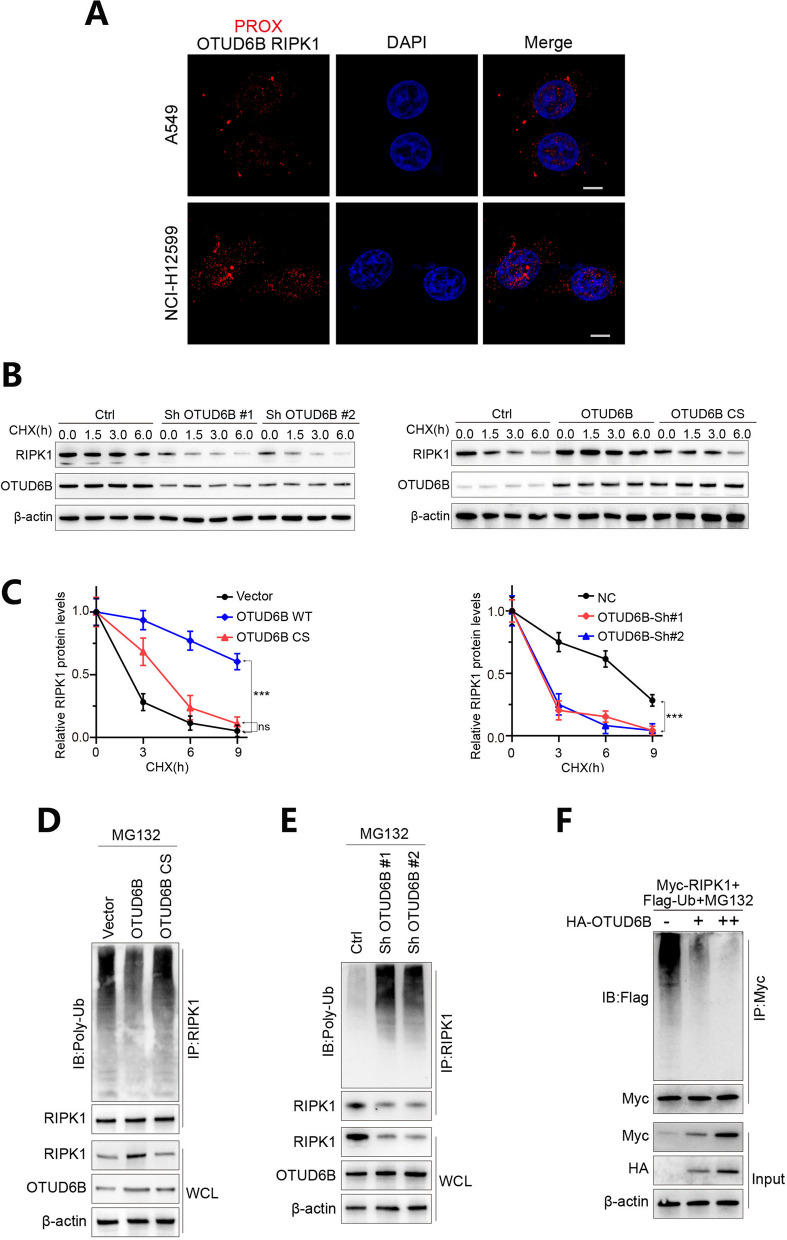
OTUD6B can bind to RIPK1, reduce its ubiquitination level and increase its protein stability. **A** The binding localization of the OTUD6B protein to RIPK1 was detected by fluorescence in situ hybridization (FISH) combined with an in situ proximity ligation assay (PLA). **B**, **C** Cycloheximide (CHX) was used to detect the effect of OTUD6B interference or mutation on the degradation rate of RIPK1 via Western blotting and qPCR. **D**–**F** The effect of OTUD6B interference on the expression level of the RIPK1 protein was verified by Western blot experiments

To investigate the specific substrate of the OTUD6B deubiquitinating enzyme, we first knocked down and overexpressed OTUD6B in LUAD cell lines and verified the results by Western blotting (Fig. 5A–B). Then, we used co-immunoprecipitation (IP) experiments to determine which proteins interact with OTUD6B in LUAD cells, and the results showed that RIPK1 can interact with OTUD6B in LUAD cells (Fig. 5C, D). Then, through verification via Western blot experiments, we found that OTUD6B regulates the expression of RIPK1 at the protein level (Fig. 5E, F). Moreover, we used a proteasome pathway inhibitor (MG132) to evaluate the effect of interfering with OTUD6B on the degradation of RIPK1. Finally, we verified the interaction between OTUD6B and RIPK1 by performing exogenous and endogenous two-way co-immunoprecipitation (CO-IP) (Fig. 5G–K). In addition, we used fluorescence in situ hybridization (FISH) combined with an in situ proximity ligation assay (PLA) to detect the binding localization of the OTUD6B protein to RIPK1 (Fig. 6A). Cycloheximide (CHX) was used to detect the effect of OTUD6B interference on the degradation rate of RIPK1 and the effect of OTUD6B mutation on the degradation rate of RIPK1 (Fig. 6B, C); moreover, the effect of OTUD6B interference on the expression level of the RIPK1 protein was verified by Western blot experiments (Fig. 6D–F). These results indicated that OTUD6B can bind to RIPK1, reduce its ubiquitination level and increase its protein stability.

### Upregulation of RIPK1 can partially reverse the cytostatic phenotype induced by knockdown of OTUD6B

**Fig. 7:**
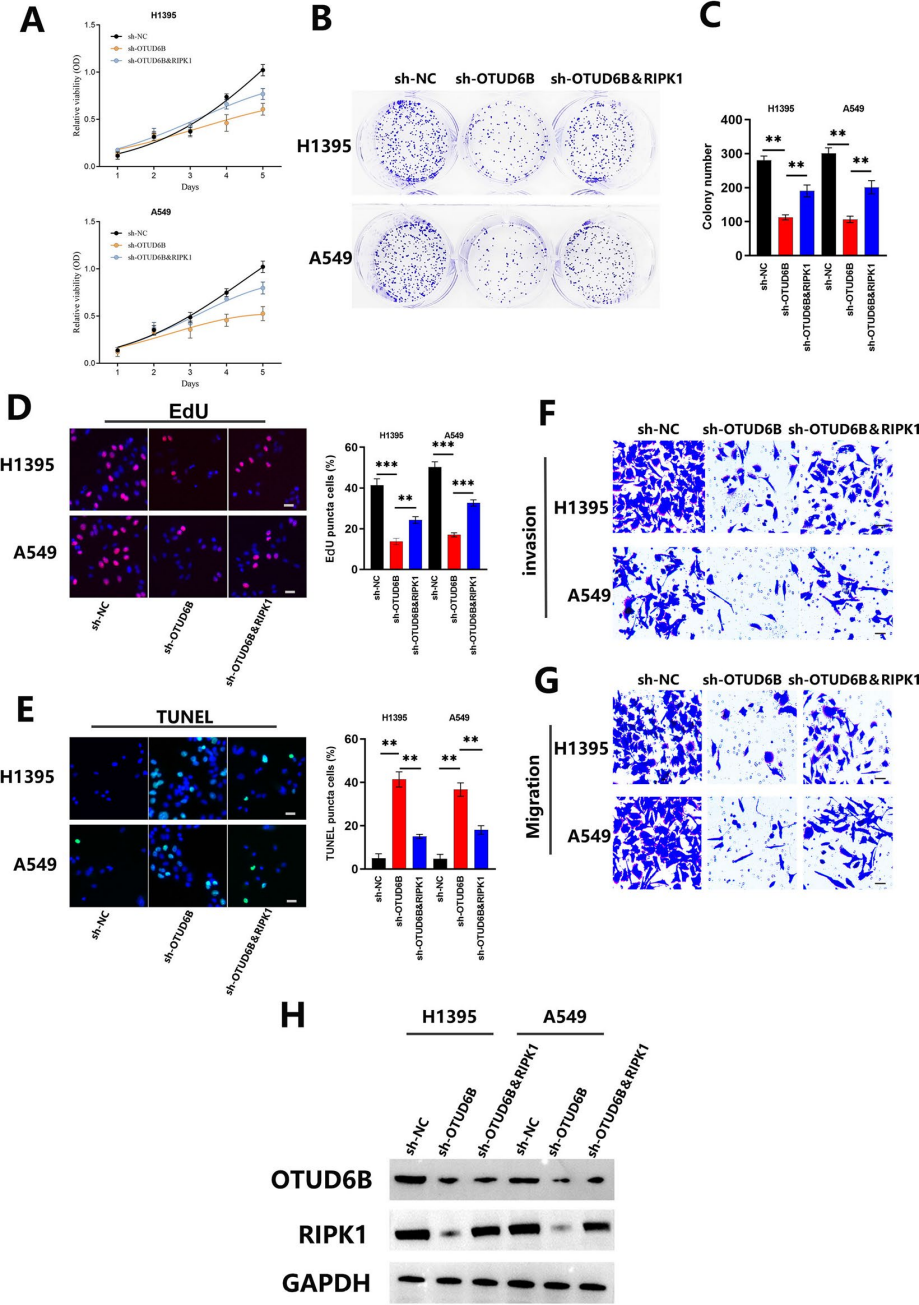
Upregulation of RIPK1 can partially reverse the cytostatic phenotype induced by knockdown of OTUD6B. **A**–**D** The proliferation ability of LUAD cell lines with shNC, shOTUD6B, OTUD6B knockdown and RIPK1 overexpression (shOTUD6B&RIPK1) was examined by CCK8 proliferation, plate colony formation and EdU cell proliferation assays. **E** TUNEL staining was used to detect apoptosis in LUAD cell lines. **F**, **G** The metastasis ability of LUAD cell lines was examined by invasion and migration assays. **H** The protein expression levels of OTUD6B and RIPK1 were detected by Western blotting in LUAD cell lines with OTUD6B knockdown and RIPK1 overexpression

To verify the influence of the OTUD6B/RIPK1 regulatory axis on the malignant biological behavior of LUAD cells, we conducted experiments in vitro. We constructed a RIPK1 protein expression complement LUAD cell line (shOTUD6B&RIPK1) and detected the protein expression levels of OTUD6B and RIPK1 by Western blotting (Fig. 7H). Then, we used a CCK-8 assay to detect cell proliferation inhibition (Fig. 7A), plate colony formation analysis (Fig. 7B, C), an EdU staining test to detect cell proliferation (Fig. 7D), TUNEL staining to detect the cell apoptosis rate (Fig. 7E), and a transwell test to detect cell migration and invasion ability (Fig. 7F, G). The results showed that the changes in cell proliferation, cell apoptosis and cell metastasis caused by OTUD6B silencing could be partially reversed by restoring RIPK1 protein expression in LUAD cell lines.

### OTUD6B and RIPK1 expression levels are positively correlated in LUAD

**Fig. 8:**
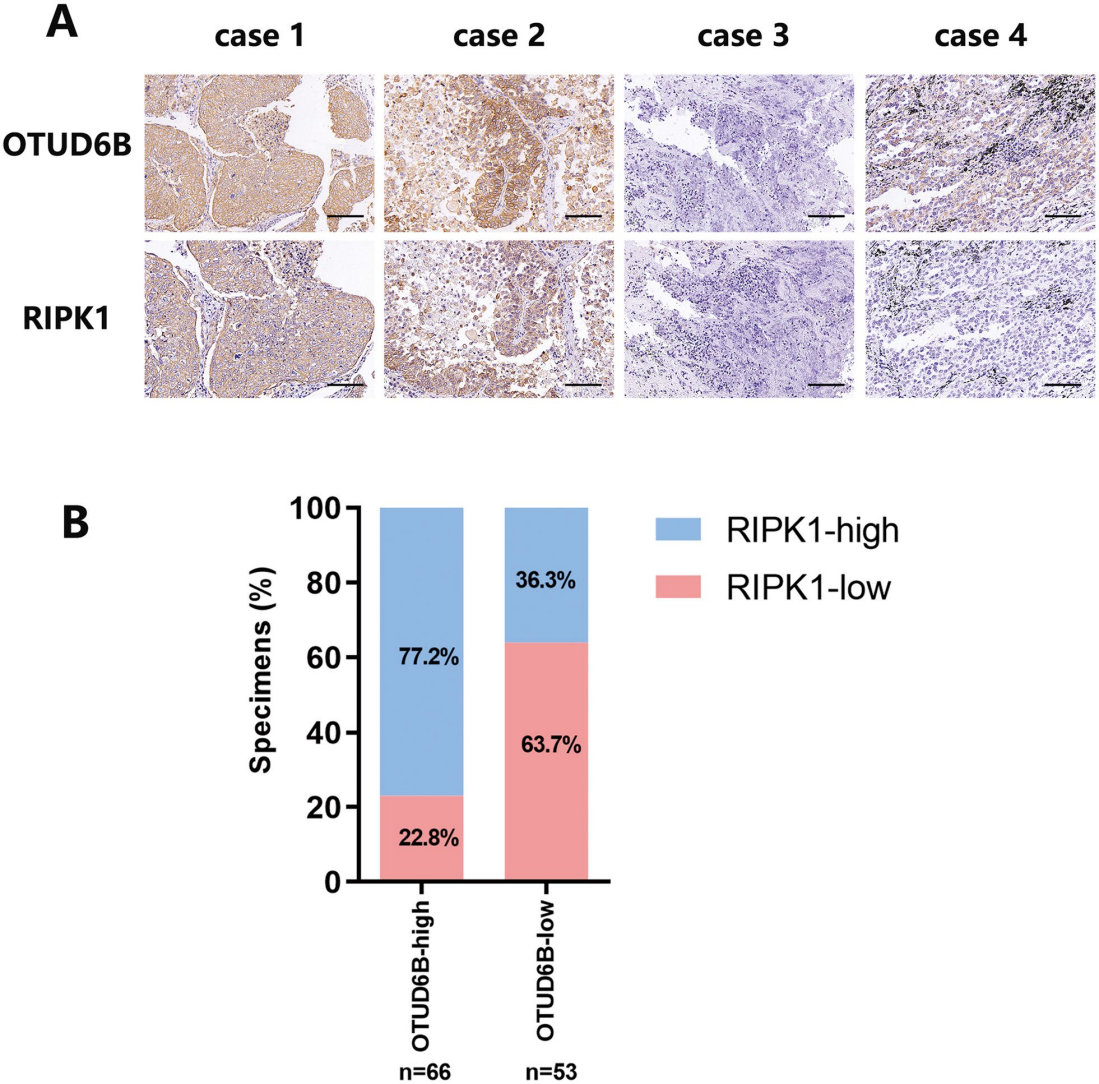
There was a positive correlation between OTUD6B and RIPK1 expression levels in LUAD. **A**, **B** The expression levels of RIPK1 and OTUD6B in the cancer tissues of 119 patients with LUAD were detected via immunohistochemistry, and the correlations between them were statistically analyzed

We used immunohistochemistry to detect the expression levels of RIPK1 and OTUD6B in the cancer tissues of 119 patients with LUAD and analyzed the correlation between them by statistical methods (Fig. 8A, B). The results showed that the expression levels of OTUD6B and RIPK1 were positively correlated in LUAD tissues.

## Discussion

OTU family members have been shown to regulate the stability and activity of several proteins involved in cell signaling, thereby playing an important role in tumor progression. However, the role of OTUD6B in LUAD has not been reported. In addition, the regulatory role of OTUD6B as a deubiquitinating enzyme in LUAD is also unknown. Therefore, research on OTUD6B in LUAD is necessary.

OTUD6B is a member of the deubiquitinase family and can play an important role in the occurrence and development of various diseases. The expression of OTUD6B has been reported to have a significant impact on tumor progression. For example, related reports have demonstrated that deubiquitinase OTUD6B isoforms are important regulators of growth and proliferation [17]. Inactivation of OTUD6B almost completely eliminates the growth of multiple myeloma (MM) in vivo through cell cycle arrest at the G1/S checkpoint, which is consistent with the associated loss of MYC expression [14]. As shown in our supplementary material, the expression of G1/S phase-related cycle proteins was also verified by Western blot, and the results suggested that the knockdown of OTUD6B could significantly inhibit G1/S phase cycle arrest. In addition, in triple-negative breast cancer, OTUD6B can activate autophagy and DDR inhibition through the OTUD6BAS1/miR-26a-5p/MTDH axis, thereby mediating genomic instability and promoting the development of PTX drug resistance [18]. The deubiquitinase OTUD6B inhibits cell migration in clear cell renal cell carcinoma and hepatocellular carcinoma by stabilizing mutated pVHL [16]. Lentivirus-mediated overexpression of OTUD6B-AS1 significantly reduces cell proliferation and promotes cell apoptosis in clear cell renal cell carcinoma (ccRCC), while OTUD6B-AS1 overexpression of β-catenin reduces the activity of the Wnt/β-catenin pathway and partially inhibits cell migration and invasion [19]. This finding is consistent with our verification in this study. In addition, recent studies have shown that OTUD6B is not only highly expressed in various cancers but also identified and verified that it can be used as a potential predictive biomarker for breast cancer through bioinformatics methods [20]. At the same time, there are also studies through pancancer analysis that identify OTUD6B as a novel prognostic and immunotherapy biomarker [21]. Finally, OTUD6B also plays an important role in several diseases other than tumors; for example, biallelic variants in OTUD6B lead to mental retardation syndromes associated with seizures and dysmorphic features [22]; OTUD6B-AS1 is also a key regulator of apoptosis in systemic sclerosis [23]. Although OTUD6B has been reported to promote tumor progression, the specific substrate OTUD6B, a member of the deubiquitinase family, has not been studied. Therefore, in this paper, we verified through detailed experiments that OTUD6B can stabilize the expression of RIPK1, thereby promoting the progression of LUAD.

Receptor interacting serine/threonine kinase 1 (RIPK1) is a protein that plays a key role in the regulation of cell death and inflammation. It is a member of the RIP kinase family and is involved in several signaling pathways [24, 25]. RIPK1 is known to be involved in regulating necrosis, a type of programmed cell death triggered by certain stimuli, such as infection or tissue damage [26]. In addition, RIPK1 participates in the regulation of apoptosis, another form of programmed cell death. Mutations in the RIPK1 gene have been associated with a variety of diseases, including autoimmune and neurodegenerative disorders. Inhibition of RIPK1 has emerged as a potential therapeutic strategy for treating these diseases. In general, RIPK1 is an important protein involved in the regulation of various signaling pathways involved in cell death and inflammation, and its dysregulation is associated with various disease processes [27–29]. In our study, the interaction between the deubiquitinating enzyme OTUD6B and RIPK1 was detected by IP mass spectrometry, and the interaction between OTUD6B and RIPK1 was subsequently evaluated via endogenous and exogenous co-IP. Finally, an increase in the protein degradation rate confirmed that OTUD6B can bind to RIPK1, reduce its ubiquitination level and increase its protein stability. Immunohistochemical staining of human LUAD tissues and adjacent tissues revealed that OTUD6B and RIPK1 were highly expressed and positively correlated in LUAD. We complemented the expression data of RIPK1 in LUAD cell lines with low OTUD6B expression in vitro and found that upregulation of RIPK1 can partially reverse the cytostatic phenotype caused by OTUD6B silencing. These findings also provide strong evidence that the deubiquitinated protein OTUD6B promotes the malignant progression of LUAD by increasing the stability of the RIPK1 protein.

## Conclusions

In summary, this study demonstrated that targeting the deubiquitinating enzyme OTUD6B and its interaction with RIPK1 are potential therapeutic strategies for LUAD. By stabilizing the protein level of RIPK1, OTUD6B promotes the progression of LUAD. These findings highlight the importance of studying the regulatory roles of deubiquitinases in cancer progression and suggest that targeting the OTUD6B/RIPK1 axis may provide new opportunities for cancer therapy. However, further research is needed to better understand the molecular mechanisms underlying this interaction and to explore its potential therapeutic applications.

### Supplementary Information

**Additional file 1.**

## Data Availability

The data that support the findings of this study are available from the corresponding author upon reasonable request.
