# Supplementary material for: The deubiquitinating protein OTUD6B promotes lung adenocarcinoma progression by stabilizing RIPK1
Source: Biol Direct. 2024 Jun 16;19:46. doi: 10.1186/s13062-024-00489-8 (PMC11181667; doi:10.1186/s13062-024-00489-8)

Figure 1A:β-actin

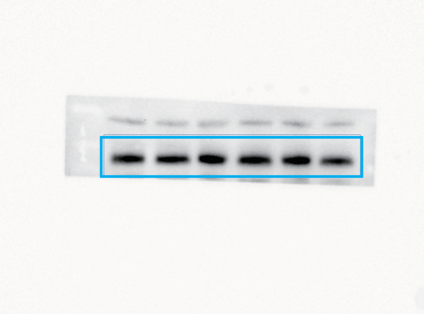

Figure 1A:OTUD6B

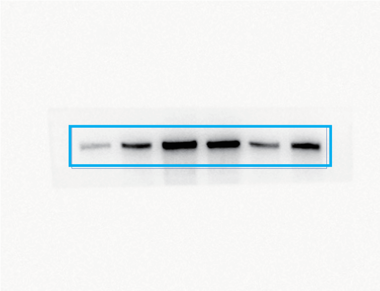

Figure 1B:β-actin

Figure 1B:OTUD6B

1-6

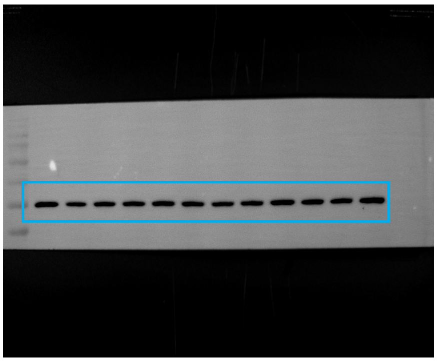

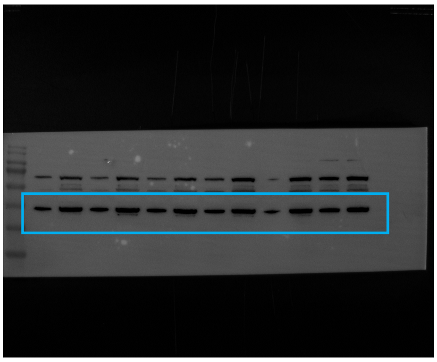

7-12

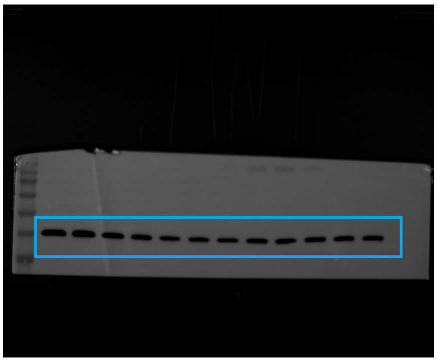

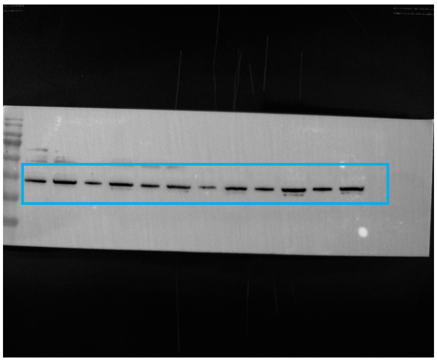

13-18

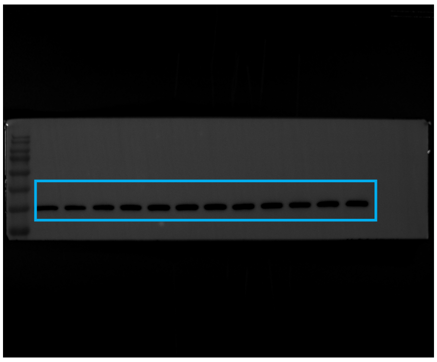

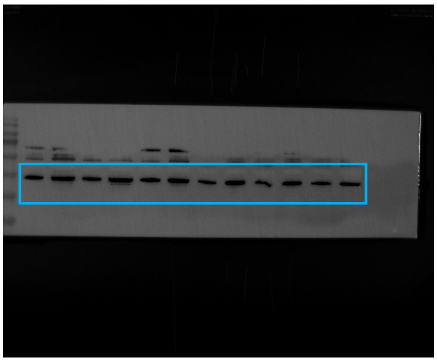

Figure 2B:β-actin

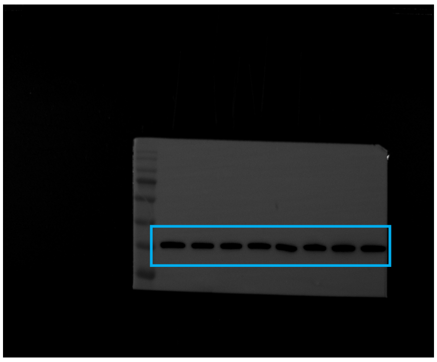

Figure 2B:OTUD6B

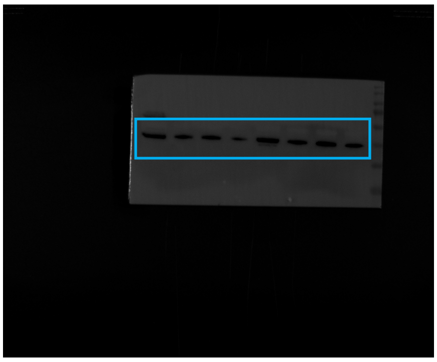

Figure 5A:β-actin

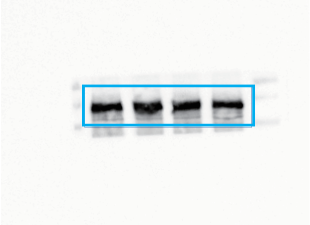

Figure 5A:OTUD6B

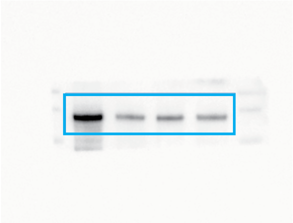

Figure 5B:β-actin

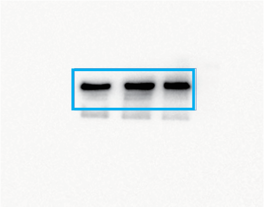

Figure 5B:OTUD6B

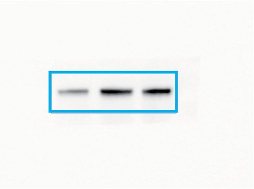

Figure 5D:β-actin

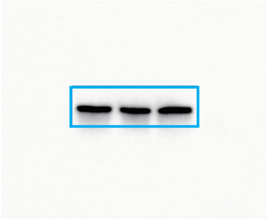

Figure 5D:HA-OTUD6B

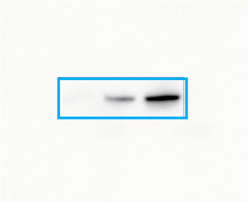

Figure 5D:Myc-RIPK1

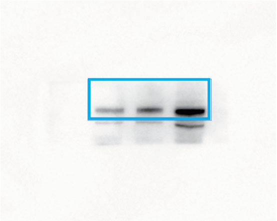

Figure 5E:β-actin

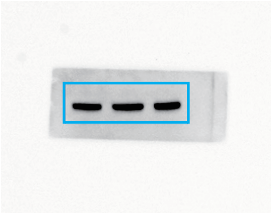

Figure 5E:OTUD6B

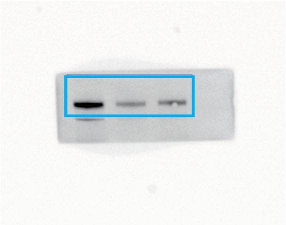

Figure 5E:RIPK1

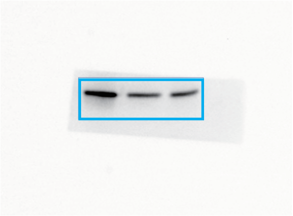

Figure 5F:β-actin

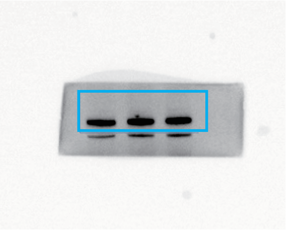

Figure 5F:OTUD6B

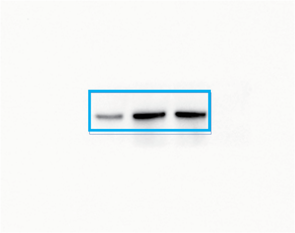

Figure 5F:RIPK1

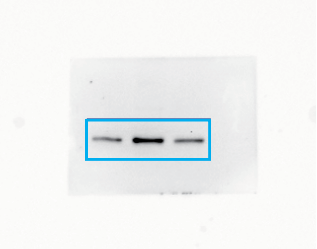

Figure 5G:β-actin

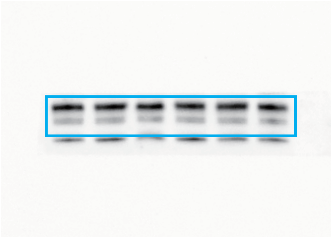

Figure 5G:OTUD6B

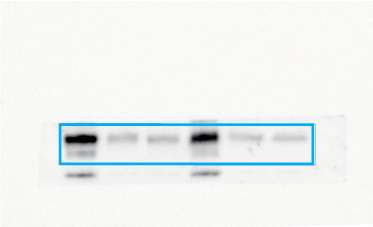

Figure 5G:RIPK1

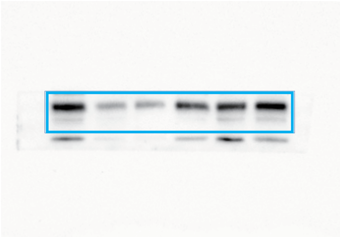

Figure 5H:HA-OTUD6B

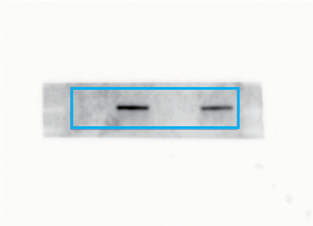

Figure 5H:Myc-RIPK1

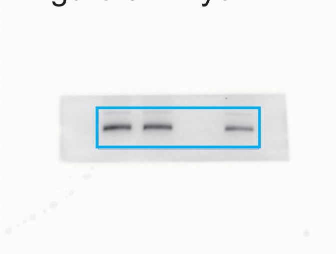

Figure 5I:Myc-RIPK1

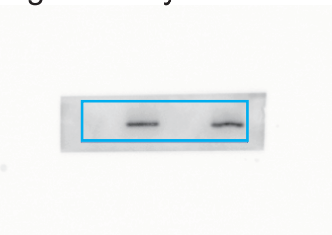

Figure 5I:HA-OTUD6B

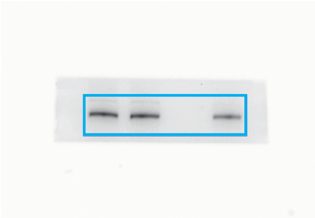

Figure 5J:RIPK1

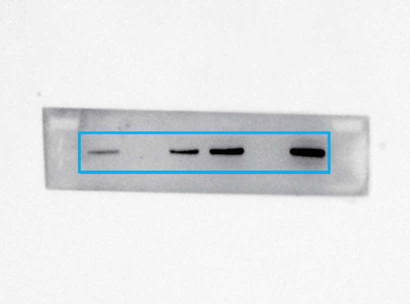

Figure 5J:OTUD6B

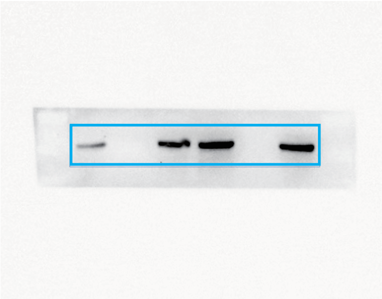

Figure 5K:RIPK1

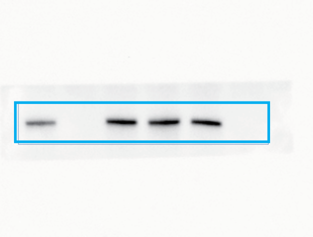

Figure 5K:OTUD6B

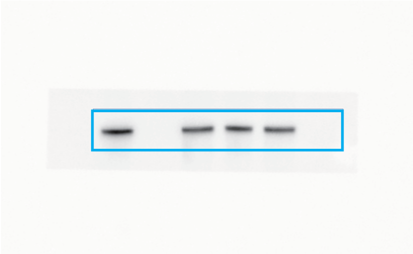

Figure 6B左图:β-actin

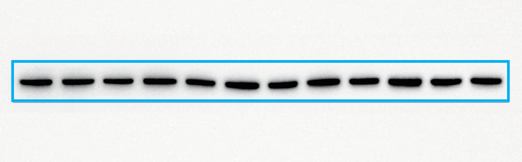

Figure 6B左图:OTUD6B

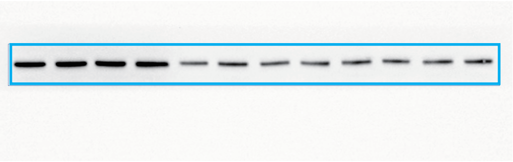

Figure 6B左图:RIPK1

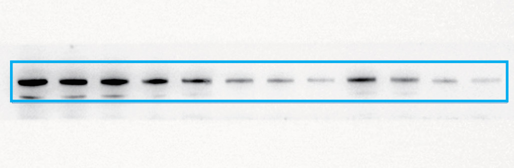

Figure 6B右图:β-actin

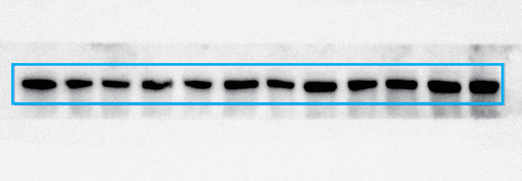

Figure 6B右图:OTUD6B

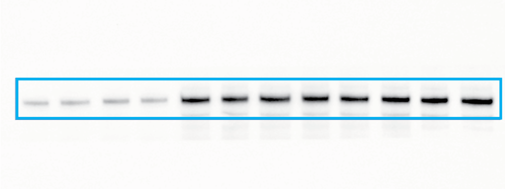

Figure 6B右图:RIPK1

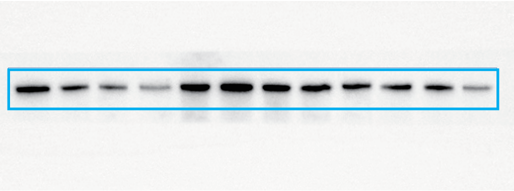

WCL

Figure 6D:β-actin

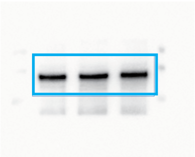

Figure 6D:OTUD6B

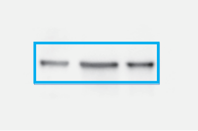

Figure 6D:RIPK1

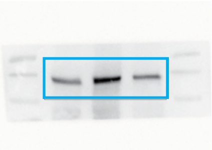

IP:RIPK1

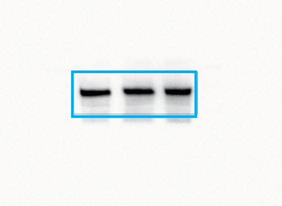

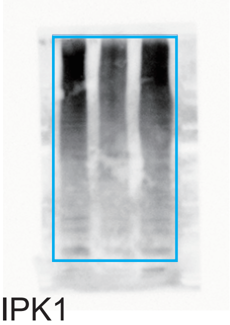

WCL

Figure 6E:β-actin

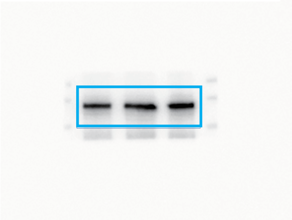

Figure 6E:OTUD6B

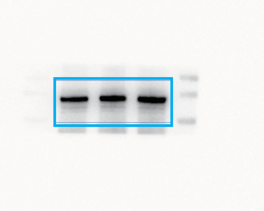

Figure 6E:RIPK1

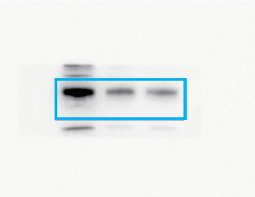

IP:RIPK1

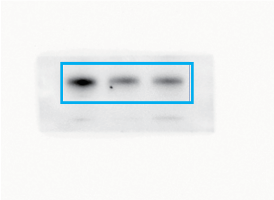

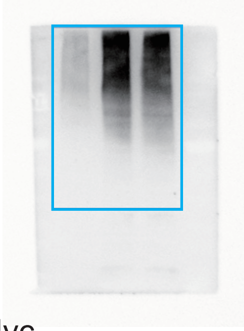

Input

Figure 6F:β-actin

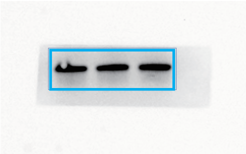

Figure 6F:HA

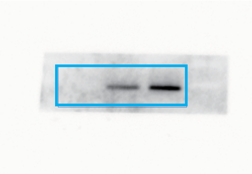

Figure 6F:Myc

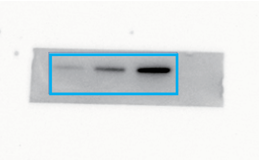

IP:Myc

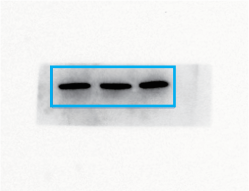

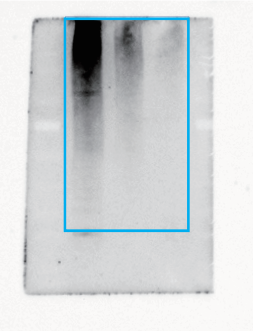

Figure 6H:GAPDH

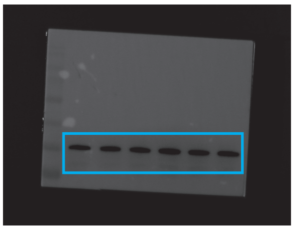

Figure 6H:OTUD6B

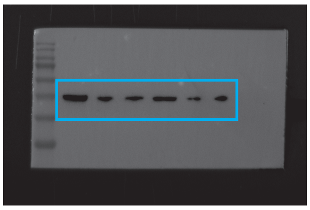

Figure 6H:RIPK1

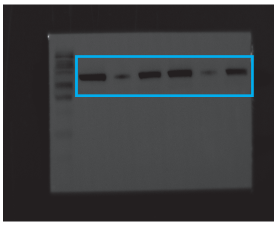

Supplement: Supplementary file 1 — Additional file 1. [file 13062_2024_489_MOESM1_ESM.pdf]
